# An *In Vivo* Fluorescence Image Analysis Tool for Esterase Activity Quantification in *Daphnia*: Using Calcein AM in Ecotoxicological Studies

**DOI:** 10.1021/acs.est.5c03309

**Published:** 2025-08-21

**Authors:** Amira Perez-Liñan, Cedric Abele, Paula Pierozan, Magnus Breitholtz, Oskar Karlsson

**Affiliations:** † Science of Life Laboratory, Department of Environmental Science, 7675 Stockholm University, 114 18 Stockholm, Sweden; ‡ Department of Environmental Science, Stockholm University, 114 18 Stockholm, Sweden

**Keywords:** calcein AM, *Daphnia*, ecotoxicology, high-content screening, fluorescence imaging

## Abstract

There is an increasing need for new approach methodologies (NAMs) to generate relevant ecotoxicological data. This study demonstrates the strengths of calcein AM, a highly sensitive fluorescent stain for esterase activity, in an automated image-based multiwell plate assay for detecting sublethal effects in *Daphnia magna*. Sample processing and feeding conditions were optimized to ensure a uniform dye distribution. The protocol was validated using two esterase inhibitors, triphenyl phosphate and netilmicin sulfate, and subsequently applied to test the environmental contaminants methoxychlor, lindane, tributyltin chloride, pentachlorophenol, diuron, and ethofumesate. The test organisms were imaged *in vivo* using automated confocal microscopy, and fluorescence intensity was quantified to generate concentration–response curves. The effects of triphenyl phosphate and netilmicin sulfate were observed at concentrations 3-fold and 6-fold lower, respectively, than in the OECD 202 immobilization test. All tested contaminants also inhibited esterase activity, with concentrations resulting in no esterase activity at 48 h, correlating with mortality observed at 48 h. This method provides a new sensitive fluorescent tool for detecting sublethal chemical effects in *D. magna*, with the added advantage of visualizing intracellular processes *in vivo*.

## Graphical abstract

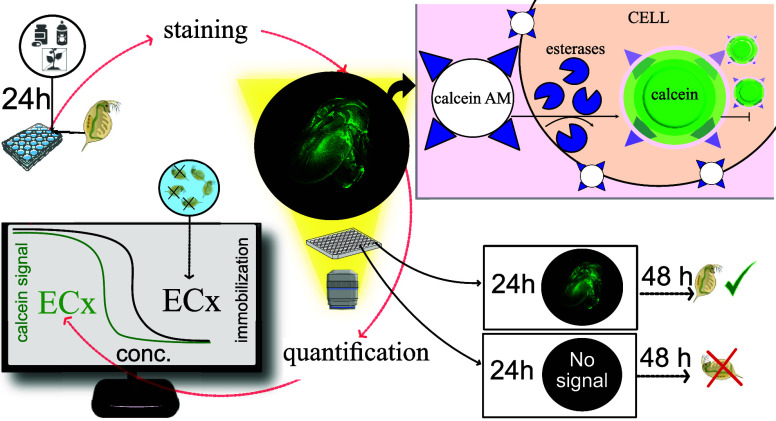

## 1 Introduction

Each year, more than 86 million tons of environmentally hazardous chemicals are produced in Europe,^1^ while thousands of new chemicals are registered globally.^2^ However, the majority of chemicals in global industrial inventories remain poorly characterized in terms of environmental toxicity.^2,3^ Consequently, there is a growing need for new approach methodologies (NAMs) that can generate high-quality ecotoxicological data in a more time and cost-efficient manner.^4^ Such methodologies have the potential to facilitate more predictive and mechanistic ecotoxicological assessments compared with traditional standard tests.

*Daphnia magna* is a keystone species commonly used in ecotoxicological research due to its alignment with the 3R’s principle, low cost, and rapid testing capabilities.^5^ Standard test methods, such as OECD guidelines, primarily assess reproductive ability (test no. 211) or median lethal concentration (test no. 202) but do not detect early biochemical responses to chemical exposure at a cellular level or elucidate mechanisms of toxicity.^6^

To address these limitations, fluorescence image-based methods are emerging as tools for predictive ecotoxicology.^7–11^ In these methods, biological samples stained with fluorescent dyes are imaged using automated microscopy and analyzed by an image analysis software.^12,13^ Advances in molecular dyes, microscope technology, and image analysis have made these methods more accessible and valuable for ecotoxicology research. Key advantages include time efficiency, spatial data collection, and quantitative analysis.^14^ Esterase enzyme activity is a recognized marker of cellular health and may serve as a valuable ecotoxicological end point.^15^ The highly lipophilic molecular stain calcein acetoxymethyl ester (calcein AM) becomes fluorescent after intracellular esterase-mediated hydrolysis and is frequently used to assess cellular viability.^16,17^

To date, only a few studies have used calcein AM staining in *D. magna*, primarily in double-staining protocols to investigate the intoxication process following exposure to different bacterial strains.^18,19^ These studies demonstrated the utility of calcein AM for visualizing live tissue in *D. magna*. However, its broader application to monitor subcellular toxicity mechanisms, such as esterase activity, remains underexplored. Although esterases have been proposed as biomarkers for contaminant exposure in aquatic organisms,^20–23^ a major limitation in biomarker studies is the rapid postmortem degradation of tissues, the time elapsed between organism death and sample processing, and the number of freeze–thaw cycles during handling.^24^ These challenges highlight the need for *in vivo* methods to assess such end points. In this study, we tackle these limitations and expand the application domain of calcein AM with the aim of developing a novel, automated fluorescence image-based protocol to detect and quantify sublethal effects on esterase activity in *D. magna*. Since calcein fluorescence correlates with esterase activity, the two esterase inhibitors triphenyl phosphate and netilmicin sulfate were used as model compounds. A fluorescein diacetate assay was also employed to confirm reductions in esterase activity.^25^ Finally, the method was applied to assess the environmental contaminants methoxychlor, lindane, tributyltin chloride (TBT-Cl), pentachlorophenol, diuron, and ethofumesate, alongside the OECD test no. 202 (*Daphnia* acute immobilization test) for comparative purposes. We hypothesized that sublethal concentrations of these chemicals negatively affect esterase activity in *D. magna*.

## 2 Materials and Methods

### 2.1 Chemicals

Calcein acetoxymethyl (Calcein AM/CAS number: 148504-34-1) was obtained from Invitrogen (Oregon, USA); methoxychlor (CAS number: 72-43-5, ≥99% purity), TBT-CL (CAS number: 1461229, ≥96% purity), and fluorescein diacetate (CAS number: 596-09-8) from Sigma-Aldrich (St. Louis, USA); triphenyl phosphate (CAS number: 115-86-6, ≥99% purity) from Sigma-Aldrich (Steinheim, Germany); lindane (CAS number: 58-89-9, ≥99% purity) and ethofumesate (CAS number: 26225-796, ≥99% purity) from Sigma-Aldrich (Darmstadt, Germany); netilmicin sulfate (CAS number: 56391-57-2, ≥99% purity) from Pharmacopeia, (Maryland, USA); pentachlorophenol (CAS number: 87-86-5, ≥96% purity) from Dr. Ehrenstorfer (Augsburg, Germany); diuron (CAS number: 330-541, ≥99% purity) from PESTANAL (Darmstadt, Germany), and ethanol (≥99% purity) from VWR (Fontenay-sous-bois, France).

### 2.2 *D. magna* as Test Organism

The model organism “*D. magna* environmental pollution test strain clone 5” of the Federal Environmental Agency, Berlin, Germany, was cultured at 20 ± 1 °C in 3 L beakers containing 2 L of M7 medium (25 organisms/beaker) and a photoperiod of 16 h light: 8 h darkness. M7 was controlled at a pH of 8.2 ± 0.2 and aerated for at least 24 h prior to use. Culture medium was renewed to 50% with fresh M7 every 8 days. The microalgae *Raphidocelis subcapitata* (6.8 mL of ≈3.5 × 10^5^ cells/mL) and *Desmodesmus subpicatus* (1 mL of ≈3.5 × 10^5^ cells/mL) were provided as food three times per week. Prior to each experiment, mothers older than 2 weeks were transferred into fresh M7 so the juveniles (<24 h old) were born and exposed under no-food conditions.

### 2.3 Optimization of Calcein AM

#### 2.3.1 Sample Processing and Food Conditions

As reported in previous studies, our pilot experiments showed that calcein AM accumulated in the organism’s gut to a significant degree.^26^ In order to acquire good quality images, different food conditions prior to the staining process were tested: (i) 1 h starvation, (ii) 24 h starvation, and (iii) born under no-food conditions. Groups of 10 juveniles (<24 h old) born under different food conditions were stained as described below. The best food condition was selected based on the distribution of the dye and the quality of the images.

#### 2.3.2 Staining Concentration

Concentrations of 0.5, 1, 2, and 5 μM of calcein AM diluted in M7 under a staining time of 60 min were selected based on previous literature findings.^18,19,26^ Groups of 10 juveniles (<24 h old) born under conditions where no food was available were exposed to the respective staining concentration in 24-well plates (VWR, Radnor, PA, USA). Each well had 1 mL of staining solution, and the plate was shaken gently at 150 rpm. After 60 min, all groups were anesthetized with 5% ethanol and transferred to a 384-well plate and centrifugated at 78*g* for 2 min as described previously.^11^ The best staining concentration was selected based on the distribution of the dye and the quality of the images.

#### 2.3.3 Staining Time

Groups of 10 juveniles (<24 h old) born under conditions where no food was available were exposed to 5 μM calcein AM under different staining times: 15, 30, 60, 120, and 180 min. After their respective staining time, each group was anesthetized, transferred to a 384-well plate, and centrifugated at 78*g* for 2 min.^11^ The shortest time that could provide good quality images with the lowest variation was selected.

### 2.4 Exposure to Esterase Inhibitors Triphenyl Phosphate and Netilmicin Sulfate

The concentrations for the exposure were based on previous OECD immobilization tests (Table SI1). Only *D. magna* groups from concentrations that showed no immobilization at 24 h were selected for staining. The lowest concentration with 100% immobilization was chosen as the immobilization control, where the lowest calcein signal is expected. *D. magna* was considered immobilized according to the OECD test no. 202 immobilization concept.^27^ Netilmicin sulfate and triphenyl phosphate were used as esterase inhibitor positive controls. Netilmicin sulfate was tested at 0.9, 3.9, 7.8, 15.6, 31.2, 62.5, and 500 mg/L dissolved in M7 from a stock solution of 2000 mg/L in M7. The control was exposed to M7 medium only; triphenyl phosphate was tested at 0.3, 0.6. 1.25, 1.66, 2.5, 3.33, 5, and 10 mg/L from a stock solution of 80 mg/mL in dimethyl sulfoxide (DMSO). The vehicle control used was equivalent to 0.0125% DMSO in M7, which corresponds to the highest DMSO concentration in the triphenyl phosphate experiments.

### 2.5 Esterase Activity Using Fluorescein Diacetate Validation Test

The effects of the positive control triphenyl phosphate on esterase activity in *D. magna* were confirmed using a method previously described.^25^ First, a group of 15 juveniles was exposed to 0.3, 5, and 10 mg/L of triphenyl phosphate, as well as a vehicle control (0.0125% DMSO). After 24 h, *D. magna* individuals were collected and frozen immediately at −80 °C. The experiment was conducted in three independent replicates.

After collection, the organisms were homogenized in 0.1 M Tris–HCl buffer in a tissuelyzer (Tissuelyzer II, QIAGEN, Hilden, Germany) at 25 Hz for 2 min and centrifuged for 2 min at 10 000*g* (4 °C). The supernatant was collected, and the enzymatic activity was measured with fluorescein diacetate as a specific substrate. The protein content of each homogenate was determined using the commercial DC Protein Assay kit from BIO-RAD (California, USA). Enzyme activity was expressed as μmol/min/mg protein. The results are shown as mean ± SD, and the differences compared to control were analyzed using a one-way analysis of variance (ANOVA) followed by Dunnett′s test.

### 2.6 Exposure to Methoxychlor, Lindane, and TBT-CL and Calcein AM Staining

As model environmental contaminants, methoxychlor (2.5, 5, 10, 25, 50, 100, and 200 μg/L), lindane (0.07, 0.15, 0.30, 0.60, 1.25, 1.87, 2.50, and 10 mg/L), TBT-CL (3.12, 6.25, 12.50, 25, 50, 200 μg/L), pentachlorophenol (0.03, 0.07, 0.15, 0.30, 0.62, 1.25; 5 mg/L), diuron (0.95, 1.87, 3.75, 7.5, 15, 30, 40 mg/L), and ethofumesate (0.78, 1.56, 3.12, 6.2, 12.5, 25, 50 mg/L) were tested. All of the stock solutions were dissolved in DMSO, and then each exposure concentration was prepared in M7. The vehicle control was DMSO, corresponding to the highest concentration in each experiment: methoxychlor 0.03%, lindane 0.1%, TBT-CL 0.001%, pentachlorophenol 0.05%, diuron 0.1%, and ethofumesate 0.05%. The exposure was conducted for 24 h in 24-well plates with 10 individuals per test concentration and in a volume of 2 mL. All chemicals were tested in three independent experiments. After the exposure, the immobilization was registered, and half of the exposure solution was removed and replaced with calcein AM staining solution at 10 μM (50:50) to reach a final concentration of 5 μM. Additionally, a blank control of 10 nonstained individuals was added to account for autofluorescence if necessary. After this, the well plate was incubated in the dark for 30 min and then the organisms were anesthetized in 5% ethanol, transferred to a 384-well plate and centrifuged at 78*g* for 2 min.^11^

### 2.7 Image Acquisition

Images were acquired using a microscope ImageXpress Micro (Molecular Devices, Sunnyvale, CA, USA) in confocal mode with a 60 μm pinhole, camera binning 2, and 4× objective (Plan Apo Lambda, 0.2 NA, air, 20 mm WD). *z*-Stacks were acquired with 10 images on different *z*-levels (distance between *z*-levels: 15.9 μm). A fluorescence image was acquired on each level with an fluorescein isothiocyanate (FITC) filter set (excitation, 475/34 nm; emission, 536/40 nm) on 100% illumination power and a transmitted light image on 50% illumination power. The transmitted light images of all *z*-levels were merged into a 2D projection, which was used for segmentation. Once the staining protocol was optimized, fluorescence images were acquired with an exposure time of 50 ms.

### 2.8 Image Analysis

The acquired images were analyzed by using the MetaXpress Custom Module software (Molecular Devices, San Jose, CA, USA). A segmentation mask was created using the transmitted light images, which was later used on the fluorescent image from the FITC channel to measure the signal^11^ (Figure SI1).

This workflow averages the fluorescence intensities of pixels within the segmented mask area. To exclude overexposed pixels, an intensity threshold of 45 000 was applied in the image analysis workflow. The data with the calcein signal was exported to an Excel file and processed in GraphPad Prism Version 10.4.0. The average intensity of 10 *z*-levels on each *D. magna* image was calculated. Images with the wrong segmentation or more than one *D. magna* detected were excluded. The average of the 10 *D. magna* per concentration from the three replicates was used to calculate EC_10_ and EC_50_ with 95% confidence intervals for each compound tested using a concentration response model based on a two-parameter log–logistic function as described previously.^11,28^

The specific R-script for the dose–response modeling and the excel files with the raw data can be found on GitHub: https://github.com/CedricAbele/CalceinAM_dose-response.

## 3 Results and Discussion

In this study, we developed a fluorescence image-based protocol using calcein AM to detect and quantify sublethal effects on esterase activity in *D. magna*. After sample processing and feeding conditions were optimized to ensure uniform dye distribution, the applicability of calcein AM was demonstrated through exposure to two model esterase inhibitors and six environmentally relevant contaminants. All tested chemicals caused a reduction in esterase activity, with the absence of calcein signal at 24 h correlating with mortality observed at 48 h. This highlights the protocol’s ability to detect early sublethal effects following chemical exposure, with the added advantage of visualizing intracellular processes *in vivo*.

### 3.1 Optimization of Feeding Conditions and Calcein AM Staining

A known challenge in using calcein AM is imaging *D. magna*, which is the accumulation of the stain in the organism’s gut. Previous studies addressed this issue by using an *ex vivo* procedure in which the tissue was homogenized.^26^ While this allowed quantification of the fluorescence signal, it limited the ability to obtain *in vivo* information on toxicological processes and target organs. In this study, calcein AM was optimized for *in vivo* use in *D. magna* as an image-based tool for ecotoxicological studies. To achieve uniform dye distribution and high-quality imaging, various feeding conditions were tested.

Starved *D. magna* displayed a strong fluorescence signal localized in the gut, regardless of the starvation duration (Figure 1A,B). This accumulation is likely due to high esterase activity associated with digestion.^29–31^ In contrast, *D. magna* born under food-deprived conditions exhibited a more even calcein fluorescence distribution (Figure 1C), enabling a more accurate quantification of esterase activity across the organism.

**Figure 1:**
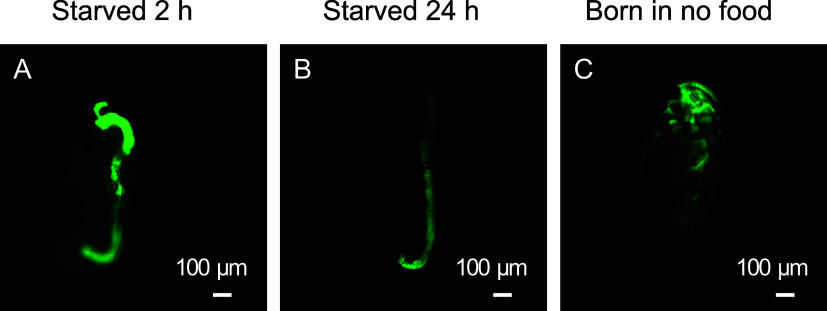
Representative calcein staining images of *D. magna* under different food conditions. (A) *D. magna* was born under normal food conditions and starved for 2 h. (B) *D. magna* starved for 24 h. (C) *D. magna* born under no-food conditions. To ensure comparability, all images are 16-bit and depicted with adjusted intensity ranges (min 4000, max 40 000).

Next, the calcein AM staining concentrations and incubation times were optimized for *in vivo* imaging. Selecting appropriate staining conditions was crucial to maximize signal intensity while minimizing variability and potential artifacts. Insufficient staining time or low concentrations produced weak fluorescence signals that impaired visualization of target structures, while excessive staining led to oversaturation, increased background fluorescence, and potential toxic effects. In our setup, fluorescence was detected at all calcein AM concentrations except 0.5μM. The signal increased with concentration, with 5 μM producing the least variability in intensity among replicates (Figure SI2), and was therefore selected as the optimal staining concentration. Fluorescence signal was detected as early as 15 min after staining and remained stable at 30, 60, and 120 min. Consequently, 30 min was chosen as the standard staining duration (Figure SI3). Once the staining protocol for calcein AM was developed, we applied it to test two esterase inhibitors and validated the observed decrease in esterase activity using a fluorescein diacetate assay.

### 3.2 *In Vivo* Calcein Signal after Exposure of *D. magna* to the Esterase Inhibitors

The effects of triphenyl phosphate exposure on *D. magna* esterase activity and immobilization were tested by using the optimized calcein staining protocol in 24-well plates. There was no difference between the OECD acute immobilization test in beakers and the immobilization measured in the 24-well plate (Table SI1). However, the EC_50_ obtained from the calcein signal was approximately 3-fold lower than that from the immobilization test (Table 1). All organisms except the positive immobilization controls were alive during image acquisition. The representative calcein signal images for vehicle control and triphenyl phosphate-exposed *D. magna* are shown in Figure 2A1–D1, with the corresponding transmitted light images in Figure 2A2–D2. In the vehicle control group, a strong calcein signal was observed, indicating high esterase activity (Figure 2A1). At 0.3 mg/L of triphenyl phosphate, the calcein signal persisted, suggesting that esterase activity remained largely unaffected (Figure 2B1). At 5 mg/L of triphenyl phosphate, the highest concentration tested without negative effects in the immobilization test, no calcein signal was detected (Figure 2C1). This indicates a significant reduction in esterase activity despite organisms remaining alive. At 10 mg/L of triphenyl phosphate, the calcein signal was also absent (Figure 2D1), consistent with the immobilization observed at this concentration. For netilmicin sulfate, the EC_50_ derived from the calcein signal after 24 h exposure was 6-fold lower than that determined by the immobilization test (Table 1 and Figure 4). The high confidence intervals for both EC_10_ and EC_50_ from the immobilization were due to the lack of data points between 62.50 and 500 mg/L. Furthermore, variability among triplicates in the calcein protocol may have resulted from a combination of biological variation and the compound’s distinct mode of action, which could influence esterase activity and dye uptake, leading to a more variable response.

**Figure 2:**
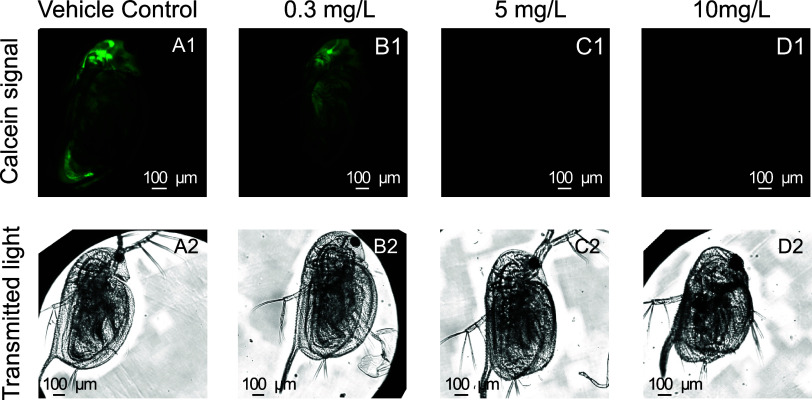
Calcein signal in *D. magna* after exposure to triphenyl phosphate. Panels (A1)–(D1) show the fluorescent calcein signal, whereas panels (A2)–(D2) show the corresponding transmitted light images. A representative *z*-layer of each image was selected for display. (A1) Vehicle control. (B1) *D. magna* exposed to 0.3 mg/L of triphenyl phosphate. (C1) *D. magna* exposed to 5 mg/L of triphenyl (the highest concentration without observed mortality). (D1) *D. magna* exposed to 10 mg/L of triphenyl phosphate (immobilization control). To ensure comparability, all images are 16-bit and intensity ranges are adjusted (min 1500, max 30 000).

**Table 1:** EC10 and EC_50_ Values for *D. magna* Derived from the Immobilization Test and Calcein Signal after 24 h of Exposure to Triphenyl Phosphate, Netilmicin Sulfate, Methoxychlor, Lindane, and TBT-CL, Pentachlorophenol, Diuron and Ethofumesate^a^

chemical	EC_*x*_	end point	24 h		unit
triphenyl phosphate	EC_10_	immobilization	6.68	(−31.40 to 44.75)	mg/L
		calcein AM	1.95	(1.55–2.36)	
	EC_50_	immobilization	7.06	(−32.98 to 47.10)	
		calcein AM	2.59	(2.36–2.82)	
netilmicin sulfate	EC_10_	immobilization	149.79	(−3281.92 to 3581.50)	μg/L
		calcein AM	4.71	(−10.91 to 20.32)	
	EC_50_	immobilization	175.84	(−3843.45 to 4195.13)	
		calcein AM	29.72	(0.12–59.32)	
methoxychlor	EC_10_	immobilization	133.27	(−388.98 to 655.52)	μg/L
		calcein AM	21.89	(4.40–39.39)	
	EC_50_	immobilization	141.55	(−402.40 to 685.50)	
		calcein AM	28.07	(8.86–47.28)	
lindane	EC_10_	immobilization	4.61	(−423.49 to 432.71)	mg/L
		calcein AM	0.61	(0.29–0.93)	
	EC_50_	immobilization	5.01	(−453.73 to 463.76)	
		calcein AM	1.24	(0.96–1.53)	
TBT-Cl	EC_10_	immobilization	91.22	(−3293.95 to 3476.41)	μg/L
		calcein AM	23.88	(6.09–41.65)	
	EC_50_	immobilization	100.19	(−3565.07 to 3765.45)	
		calcein AM	36.15	(19.50–52.81)	
pentachlorophenol	EC_10_	immobilization	2.27	(−71.93 to 76.48)	mg/L
		calcein AM	0.57	(0.26–0.88)	
	EC_50_	immobilization	2.50	(−78.26 to 83.26)	
		calcein AM	0.86	(0.54–1.19)	
diuron	EC_10_	immobilization	33.67	(13.21–54.12)	mg/L
		calcein AM	6.93	(1.61–12.25)	
	EC_50_	immobilization	34.74	(14.05–55.43)	
		calcein AM	13.96	(9.27–18.64)	
ethofumesate	EC_10_	immobilization	33.40	(−192.17 to 259.02)	mg/L
		calcein AM	21.44	(−14.14 to 57.03)	
	EC_50_	immobilization	35.30	(−201.86 to 272.48)	
		calcein AM	26.57	(8.13–45.01)	

a The data are derived from three independent experiments, with each individual data point representing the average of 10 replicates.

These results demonstrate the sensitivity of calcein AM staining for detecting early biochemical responses to chemical exposure. Beyond its established role as a live/dead stain,^17^ the absence of calcein signal at sublethal concentrations underscores its potential as a more sensitive end point than traditional immobilization tests.

To confirm that the decrease in the calcein signal reflected reduced esterase activity, a fluorescein diacetate assay was conducted. The signal from control organisms indicated high esterase activity, whereas *D. magna* exposed to the highest triphenyl phosphate concentration (immobile organisms) showed no signal. Notably, in contrast to the *in vivo* calcein imaging results, the fluorescein diacetate assay performed on homogenized organisms showed a significant decrease in the signal already at 0.3 mg/L of triphenyl phosphate. However, at 5 mg/L, the signal did not differ significantly from that of the immobilization control (Figure 3). This discrepancy may be explained by the multixenobiotic resistance (MXR) system in *Daphnia* spp., which is mediated by membrane-based transport proteins that actively export a wide range of chemicals from the cell.^26,32,33^ Calcein AM is a known substrate of MXR transporters and is commonly used in cellular dye efflux assays. After cellular uptake, calcein AM is hydrolyzed by intracellular esterases into fluorescent calcein, which is not a substrate for MRX transporters. Thus, cells with an active MXR system accumulate low levels of fluorescent calcein, whereas increased fluorescence indicates MXR inhibition.^34^ This mechanism may explain the lower fluorescence signal observed at 0.3 mg/L of triphenyl phosphate in the fluorescein diacetate assay, suggesting that the assay primarily reflects triphenyl phosphate-mediated esterase inhibition. In contrast, when using calcein AM, triphenyl phosphate may simultaneously inhibit both esterase activity and the MXR transport system. At low triphenyl phosphate concentrations, partial MXR inhibition may increase the level of intracellular calcein retention, increasing the signal slightly. At higher concentrations, strong esterase inhibition likely prevents calcein AM conversion, resulting in reduced fluorescence despite MXR inhibition.

**Figure 3:**
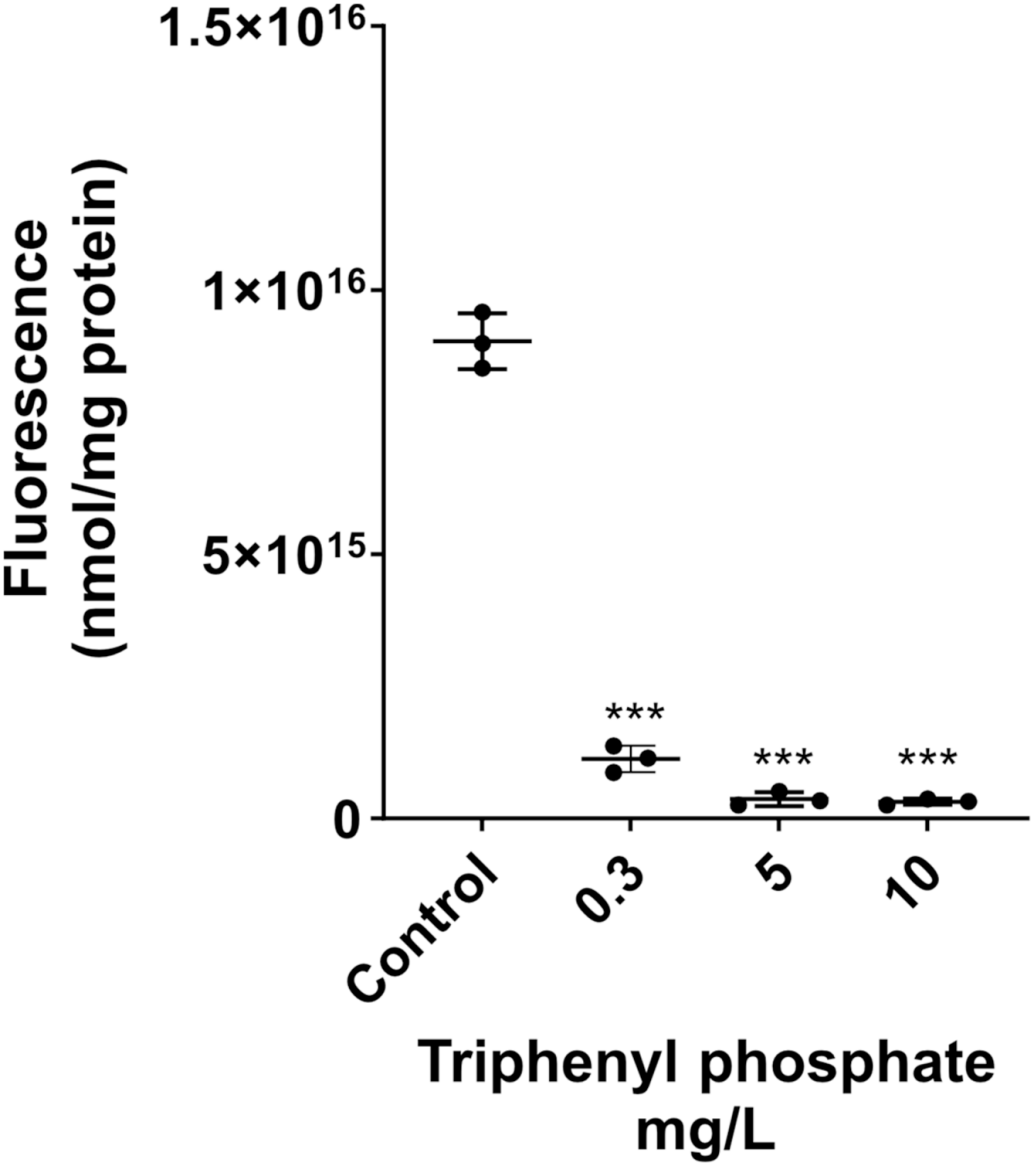
Effects on esterase activity in *D. magna* after 24 h exposure to 0.3, 5, and 10 mg/L of triphenyl phosphate. Values represent the mean ± standard deviation (SD) from three independent experiments, with 15 individuals per concentration. Asterisks indicate significant difference from control (ANOVA *P* < 0.0001).

The fluorescein diacetate assay required more organisms per concentration and was more time-consuming than the image-based calcein method. A major limitation in biomarker-based assays is the rapid postmortem tissue degradation and variability introduced during sample handling,^35^ which can directly affect the accuracy of the results. These limitations were evident when using fluorescein diacetate but were effectively mitigated using our image-based calcein AM. This latter method more accurately reflects the intracellular process *in vivo*. Furthermore, the semi-automated imaging and analysis pipeline enables rapid data acquisition and can identify if particular organs or tissues are more sensitive to chemical exposure.

After confirmation of the suitability of calcein AM for detecting esterase inhibition by the model compounds, the protocol was applied to assess six environmentally relevant contaminants.

### 3.3 Calcein Signal after Exposure to Environmental Contaminants

Given the close phylogenetic relationship between crustaceans and insects, insecticides often pose a significant hazard to species such as *D. magna*.^36^ We therefore selected two insecticides (methoxychloride and lindane) to assess the ability of calcein AM to detect sublethal effects *in vivo*. In addition, we tested the two potent biocides TBT-CL and pentachlorophenol, which are known to harm many nontarget aquatic organisms, including *D. magna*.^37–39^ We also tested diuron and ethofumesate, two herbicides commonly detected in surface waters.^34,40^

Despite regulatory bans, organochlorine insecticides such as methoxychlor and lindane continue to be detected in surface waters.^41,42^ Methoxychlor has been linked to mortality in *D. magna* at concentrations of 10 μg/L and above, while behavioral and physiological changes have been observed at 2.5 μg/L.^43^ Lindane impairs growth, reproduction, and survival at concentrations of 250 μg/L or higher. It also affects swimming, behavior, feeding patterns, and metabolic processes.^36^

In our study, the EC_50_ values derived from the *in vivo* calcein signal in *D. magna*, using automated fluorescence imaging in a multiwell format, were approximately 6-fold lower for methoxychlor and 4-fold lower for lindane compared to the traditional immobilization test (Table 1 and Figure 4). Although methoxychlor and lindane are banned in Europe and other regions, they continue to be used legally or illegally in some agricultural areas.^44,45^ During its widespread use as a broad-spectrum insecticide in the United States, methoxychlor concentrations in surface waters reached up to 50 μg/L.^46^ A more recent study found methoxychlor in drinking water sources in Nigeria at concentrations up to 56 μg/L.^47^ This concentration was particularly critical in our methoxychlor exposure experiments as it resulted in a complete loss of esterase activity, despite the absence of immobilization. Interestingly, organisms exposed to concentrations that caused a loss of calcein signal after 24 h were found dead within 48 h (Tables 1 and SI1), highlighting the predictive value of esterase activity as an ecotoxicological end point. The lower EC_50_ for methoxychlor (28.07 μg/L) supports esterase activity as an early warning biomarker for sublethal toxicity. This result aligns with similar observations in other aquatic organisms, as a recent study demonstrated negative effects on reproduction and behavior in freshwater snails exposed to methoxychlor at a comparable concentration of 25 μg/L.^48^

**Figure 4:**
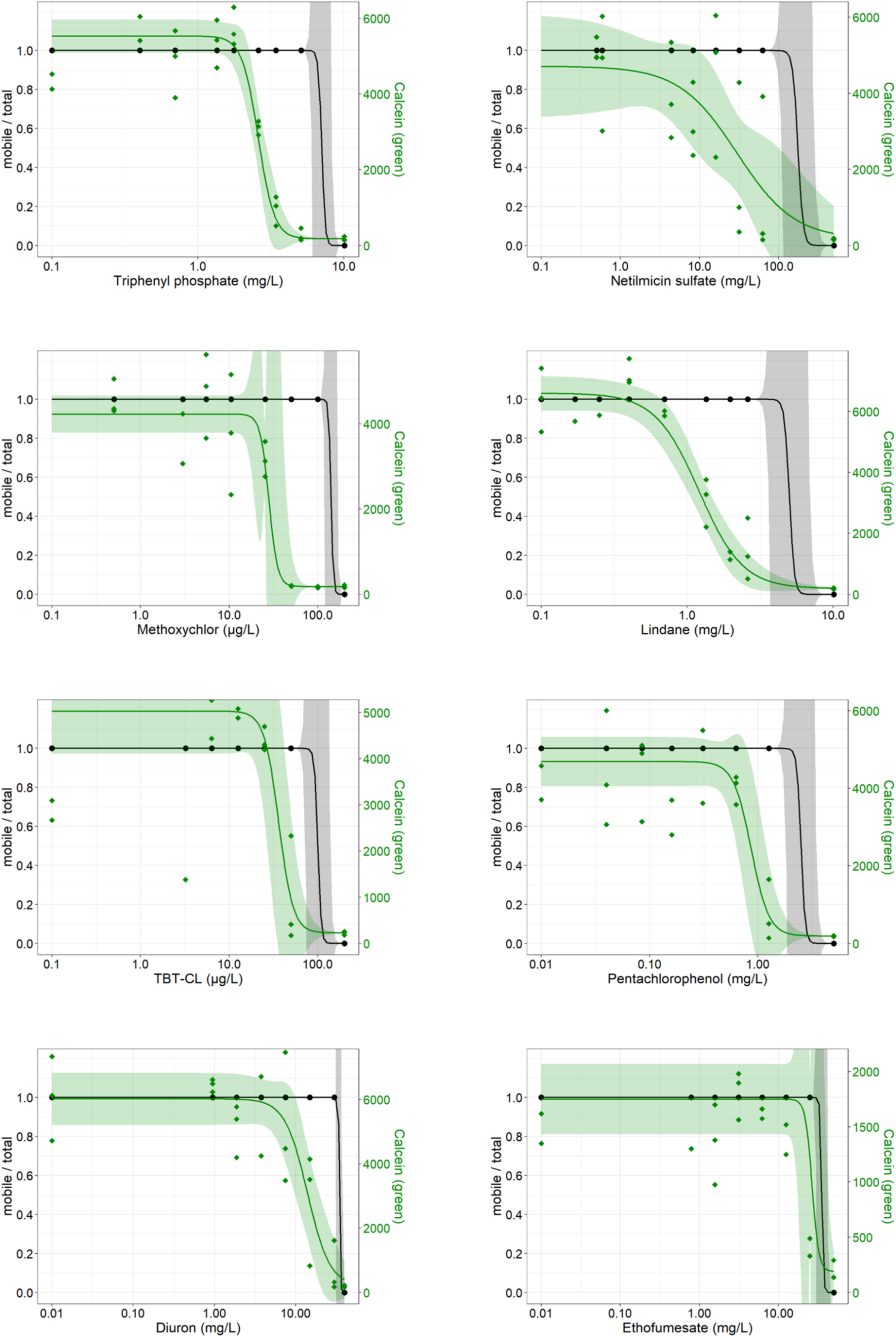
Immobilization (gray) and calcein signal (green) of *D. magna* from the image-based method after 24 h of exposure to triphenyl phosphate, netilmicin sulfate, methoxychlor, lindane, TBT-CL, pentachlorophenol, diuron, and ethofumesate. Each point represents the average of 10 technical replicates from three independent experiments. Concentrations and their respective response are shown with 95% confidence intervals.

Lindane was banned in 2008 by the European Union.^49^ However, due to its persistence in the environment, former agricultural areas and production/disposal sites remain significant contamination reservoirs. These sites continue to leach lindane into soil and water, polluting both groundwater and surface water, threatening sources of drinking water.^50,51^ For example, a 2023 study reported lindane concentrations as high as 2.60 μg/L in surface waters in Pakistan.^52^ Similar contamination has been documented across Europe, where an estimated 1.8–3 million tons of lindane waste remain. Alarmingly, the environmental footprint of these contaminated sites is expected to grow over time.^53^ Using our developed method, we determined an EC_50_ of 1.24 μg/L, which is approximately half of the concentrations found in contaminated agricultural sites. This demonstrates both the high sensitivity and environmental relevance of the method. In addition, we calculated an EC_10_ of 0.61 mg/L, which is 7.5 times lower than the EC_10_ obtained from the immobilization test (Table 1 and Figure 4). Notably, a significant reduction in *D. magna* brood production and offspring numbers have been reported at similar concentrations (0.60 mg/L).^54^

Despite the efforts to ban the trade and distribution of TBT-based antifouling paints, environmental contamination remains an issue.^55–57^ This biocide is extremely toxic to aquatic organisms. In *D. magna*, studies have reported effects on photobehavior at concentrations as low as 0.5 μg/L^37^ and reproductive effects at 1 μg/L. Previous studies have also shown that lindane and TBT-CL reduce esterase activity in *D. magna* after 24 h of exposure.^58^ In our study, we calculated an EC_50_ value (36.15 μg/L) for TBT-CL, which is three times lower than the EC_50_ obtained from the immobilization test at 24 h (Table 1 and Figure 4). Similar to other test compounds, TBT-Cl resulted in 100% mortality in 48 h at the concentration at which no calcein signal was detected at 24 h (Tables 1 and SI1). These results suggest that a lack of calcein signal after 24 h of exposure is predictive of mortality observed after 48 h of exposure.

Pentachlorophenol is not only primarily used as a wood preservative but also functions as a biocide, herbicide, insecticide, and antifouling agent. Due to its high toxicity and endocrine-disruptive effects, the EU banned its production and set a new limit value in 2021.^59^ However, this ban was not endorsed by other countries such as the US where the wood industry is allowed to use pentachlorophenol until suitable alternatives are found.^60,61^ Pentachlorophenol is toxic to aquatic organisms and in environmentally relevant concentrations (53–532 μg/L), it has been shown to affect survival, age at first reproduction, fecundity, length of mothers, and number of molts in *D. magna*.^62,63^ Our method calculated an EC_50_ of 0.86 mg/L, which is almost three times lower than the immobilization-based EC_50_. Reduced esterase activity was observed even at concentrations where organisms remained mobile, with mortality following 48 h (Table 1 and Figure 4).

Diuron is a widely used herbicide and antifouling agent. It has been globally detected at concentrations ranging from nanograms per liter to milligrams per liter in surface water,^64^ groundwater,^65^ and even drinking water.^40,66^ In *Daphnia* spp., it has been shown to impair survival and reproduction at 7.7 mg/L.^67^ Using our method, we calculated an EC_50_ of 13.96 mg/L compared to 34.74 mg/L from the immobilization test (Table 1 and Figure 4).

Ethofumesate is a currently approved herbicide that inhibits plant growth and has been found in water bodies at concentrations up to 51.1 μg/L.^68^ Ethofumesate exposure in *D. magna* significantly induces the release of undeveloped eggs at 1.9 mg/L and significantly reduces body size from 11.11 mg/L.^69^ In our method, we calculated an EC_50_ of 26.57 mg/L, which is slightly lower than the EC_50_ from the immobilization test (35.3 mg/L). Unlike the other tested compounds, ethofumesate did not cause a clear drop in esterase activity prior to immobilization, suggesting a mode of action unrelated to esterase inhibition. This also confirms previous studies where ethofumesate was found to be the least harmful herbicide for *Daphnia* spp.^70^

The high confidence intervals are due to the limited number of data points between the highest concentration tested with no observed immobilization and the lower concentration with 100% immobilization (used as a positive control). As a result, the dose–response curve for the immobilization test lacks resolution in this critical concentration range, leading to wider confidence intervals for the EC_10_ and EC_50_ values. To further support this, we have included full response curves and associated statistics (including 95% confidence intervals) in the Supporting Information for each compound, where more data points were used for the immobilization assay, resulting in narrower confidence intervals.

Altogether, these findings highlight the value of calcein AM fluorescence as a sensitive biomarker for sublethal toxicity in *D. magna*. The developed image-based method enables *in vivo* quantification of esterase activity and demonstrates sensitivity higher than that of the standard OECD Acute Immobilization Test (test no. 202) across both model esterase inhibitors and environmental contaminants, avoiding degradation-related artifacts and more accurately reflecting intracellular processes. This approach allows for the assessment of sublethal toxic effects in *D. magna*. Future research should investigate whether esterase inhibition correlates with long-term effects on other biological end points, such as behavior, reproduction, and growth. Moreover, the versatility of the method extends beyond calcein AM. We previously developed an automated, image-based fluorescence method to assess mitochondrial toxicity in *D. magna*, which also achieved greater sensitivity than the OECD immobilization test.^11^ With the increasing availability of fluorescent dyes and the potential for multichannel imaging, this method can be expanded to include additional biomarkers to offer deeper insight into the mechanisms of toxicity.

While standard fluorescence microscopy is not easily scalable, our use of multiwell plates enables simultaneous, semiautomated analysis of multiple organisms, an essential feature for high-throughput screening.

## Supplementary Material



## Notes

Image analysis, staining concentrations, staining times, 24 and 48 h acute toxicity data of triphenyl phosphate, netilmicin sulfate, lindane, methoxychlor TBT-CL, pentachlorophenol, diuron, and ethofumesate (PDF)

## Notes

The authors used Grammarly AI-powered tool and ChatGPT (OpenAI) to correct typographical errors and improve the vocabulary and readability of the manuscript. All content was subsequently reviewed and edited by the authors, who assume full responsibility for the final publication.

## Abbreviations

*D. magna*: *Daphnia magna*
TBT-CL: tributyltin chloride
