# Supplementary material for: An In Vivo Fluorescence Image Analysis Tool for Esterase Activity Quantification in Daphnia: Using Calcein AM in Ecotoxicological Studies
Source: Environ Sci Technol. 2025 Aug 21;59(34):18023–32. doi: 10.1021/acs.est.5c03309 (PMC12409896; doi:10.1021/acs.est.5c03309)
Supplement: Supplementary file 1 [file es5c03309_si_001.pdf]

# An *In Vivo* Fluorescence Image Analysis Tool for Esterase Activity Quantification in *Daphnia*: Using Calcein AM in Ecotoxicological Studies

*Amira Perez-Liñan[1], Cedric Abele[1], Paula Pierozan[1], Magnus Breitholtz[2] and Oskar Karlsson[1]**

[1] Science of Life Laboratory, Department of Environmental Science, Stockholm University 11418 Stockholm

[2] Department of Environmental Science, Stockholm University, 114 18 Stockholm Sweden

* Corresponding author

Number of pages: 6

# Table of content

## Figures

**Figure SI1.** Image analysis: Segmentation mask was created based on the transmitted light image on *D. magna*. ……………………………………………………………..……………………3

**Figure SI2**. Calcein AM signal in different concentrations after 60 min staining.…………….……3

**Figure SI3.** Calcein signal at 5µM during different staining time……………………………….……..3

## Tables

**Table SI1**. *D. magna* dose response curves, EC10 and EC50 values after 24h and 48 h exposure to triphenyl phosphate, netilmicin sulphate, lindane, methoxychlor, TBT-CL, pentachlorophenol, diruon and ethofumesate……………………………………………………………….….…………4

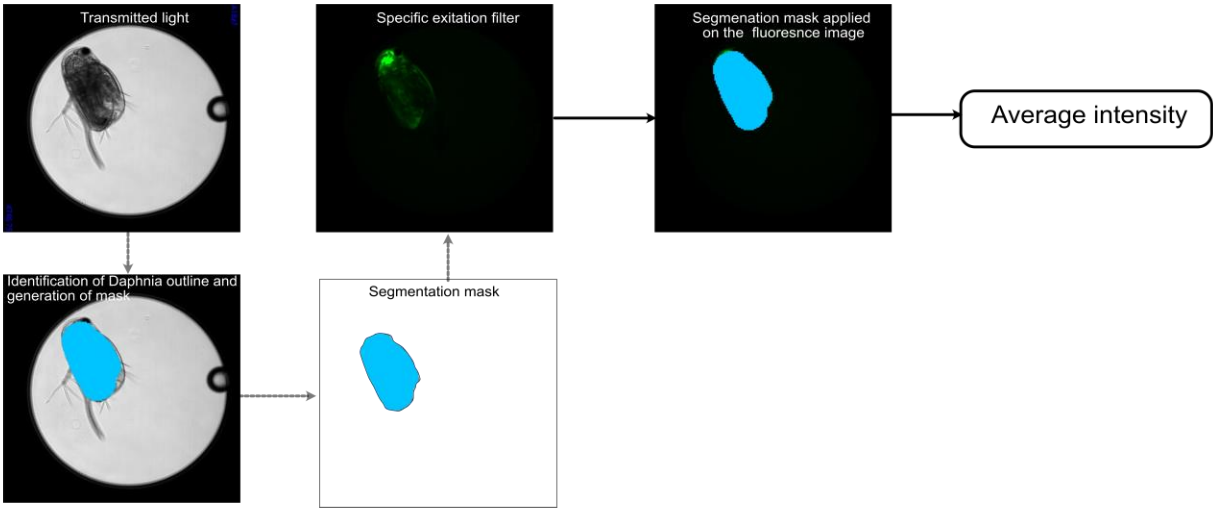


**Figure SI1**. Image analysis: A segmentation mask was created based on the transmitted light image on *D. magna*. This mask was then applied to the fluorescent image from the FITC channel to quantify the fluorescence intensity of calcein signal.

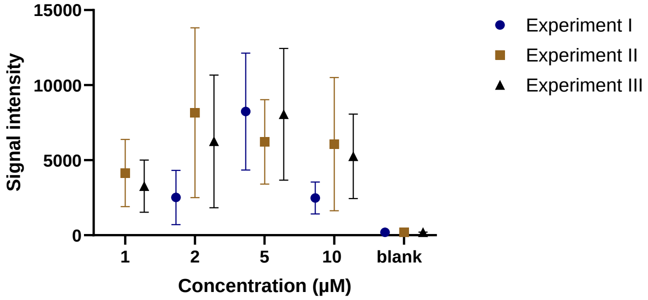


**Figure SI2**. Calcein AM signal at different concentrations after 60 minutes staining.

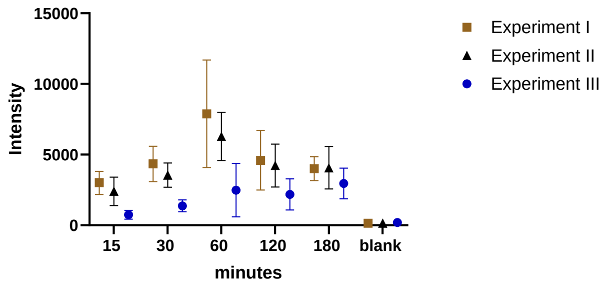


**Figure SI3.** Calcein AM signal at 5μM over different staining durations.

**Table SI1.** *D. magna* dose-response curves, EC10 and EC50 values after 24 h and 48 h exposure to TPP, netilmicin sulphate, lindane, methoxychlor, TBT-CL, Pentachlorophenol, Diuron and Ethofumesate with 95% confidence intervals.

| Chemical | Dose-response | $EC_x$ | 24 h | 48 h |
|---|---|---|---|---|
| Triphenyl Phosphate | 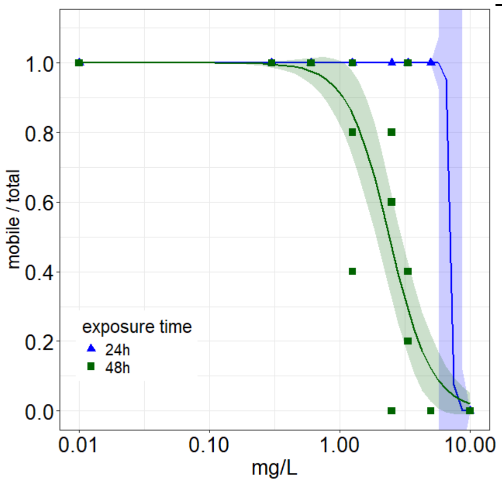  | $EC_{10}$ mg/L | 6.7(-49.8-63.18) | 1.1(0.6-1.5) |
| | | $EC_{50}$ mg/L | 7.1 (-51.7- 65.8) | 2.4 (1.9-2.9) |
| Netilmicin sulphate | 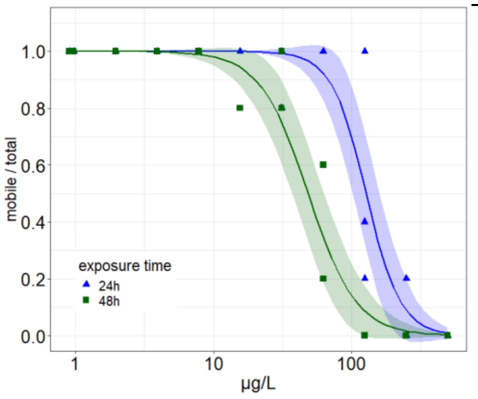  | $EC_{10}$ μg/L | 68.0 (43.6-92.4) | 20.0 (11.6-28.5) |
| | | $EC_{50}$ μg/L | 128.0 (99.2-156.8) | 48.5 (35.5-61.5) |

| | | | | |
|---|---|---|---|---|
| Methoxychlor | 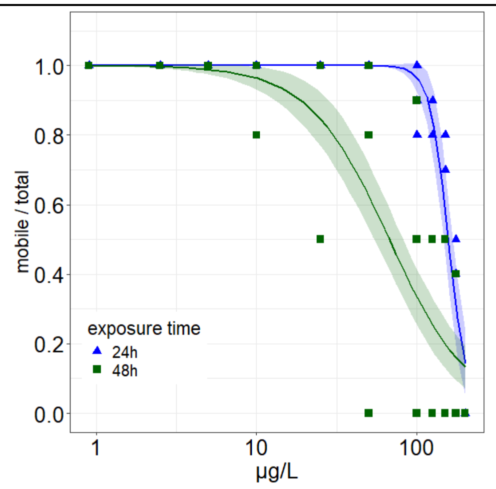 | $EC_{10}$ mg/L | 116.9 (102.9-130.9 | 18.8 (11.3-26.4) |
| | | $EC_{50}$ mg/L | 157.0 (146.8-167.2) | 67.2(53.5-80.9) |
| Lindane | 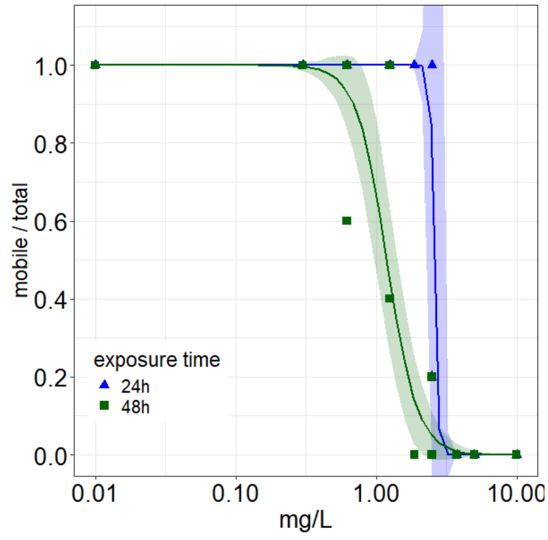 | $EC_{10}$ µg/L | 2.4 (1.6- 3.2) | 0.7(0.5-0.9) |
| | | $EC_{50}$ µg/L | 2.6 (1.8- 3.3) | 1.2(1.0-1.4) |
| TBT-CL | 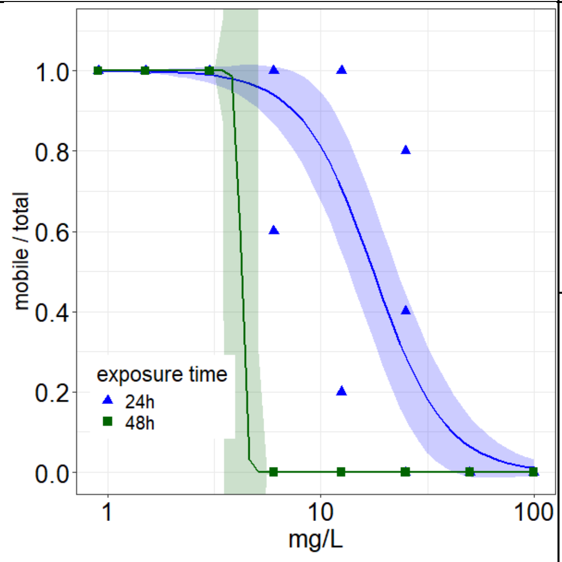 | $EC_{10}$ mg/L | 7.5 (4.3-10.7) | 4.0 (-32.3-40.3) |
| | | $EC_{50}$ mg/L | 17.6 (13.0-22.3) | 4.2 (-34.1-42.5) |

<table>
<tr><td rowspan="2">Pentachlorophenol</td><td rowspan="2">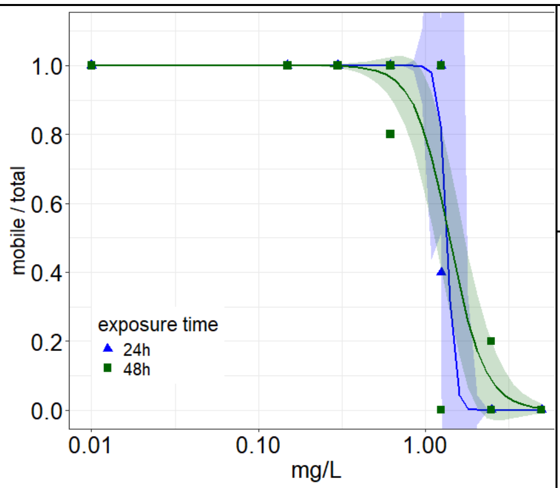
</td><td>EC10 mg/L</td><td>1.96 (0.61-1.77)</td><td>0.81 (0.55-1.09)</td></tr>
<tr><td>EC50 mg/L</td><td>1.35 (0.23-2.46)</td><td>1.33 (1.08-1.58)</td></tr>
<tr><td rowspan="2">Diuron</td><td rowspan="2">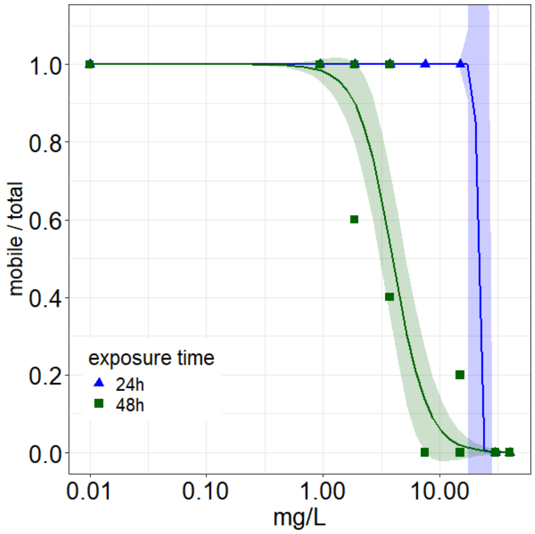
</td><td>EC10 mg/L</td><td>20.04 (-180.50-220.58)</td><td>11.81 (-17.90-41.54)</td></tr>
<tr><td>EC50 mg/L</td><td>21.15 (-191.04-233.35)</td><td>13.12 (-4.93-31.26)</td></tr>
<tr><td rowspan="2">Ethofumesate</td><td rowspan="2">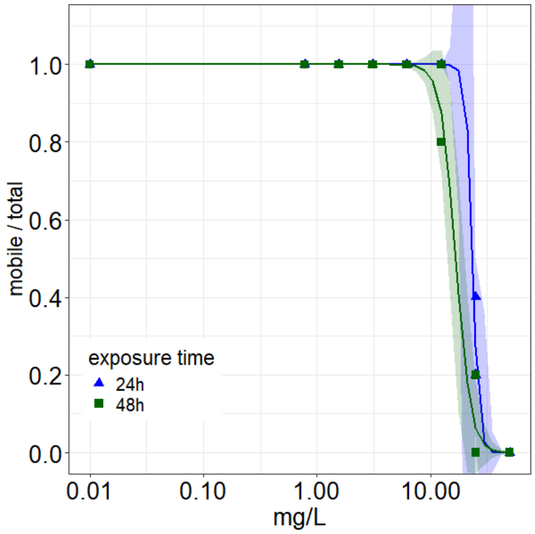
</td><td>EC10 mg/L</td><td>20.16 (-0.96-41.28)</td><td>11.96 (9.14-14.77)</td></tr>
<tr><td>EC50 mg/L</td><td>23.36 (15.48-31.23)</td><td>16.66 (13.58-19.74)</td></tr>
</table>
